# Virtual Bronchoscopy Planner and Radial-EBUS Guided Biopsy for Organizing Pneumonia Diagnosis

**DOI:** 10.3390/jcm11010104

**Published:** 2021-12-25

**Authors:** Samy Lachkar, Mathieu Salaün, Loic Perrot, Diane Gervereau, Marielle De Marchi, Gurvan Le Bouar, Helene Morisse-Pradier, Stephane Dominique, Nicolas Piton, Florian Guisier, Luc Thiberville

**Affiliations:** 1 Department of Pulmonology, Thoracic Oncology and Respiratory Intensive Care & CIC-CRB 1404, Rouen University Hospital, F-76000 Rouen, France; Loicperrot@icloud.com (L.P.); diane.gerverau@chu-rouen.fr (D.G.); Marielle.demarchi@chu-rouen.fr (M.D.M.); gurvan.lebouar@chu-rouen.fr (G.L.B.); helene.morisse-pradier@chu-rouen.fr (H.M.-P.); stephane.dominique@chu-rouen.fr (S.D.); 2 EA4108 LITIS Lab, CHU Rouen, Department of Pneumology and Inserm CIC-CRB 1404, UNIROUEN, Normandie Univ, F-76000 Rouen, France; mathieu.salaun@univ-rouen.fr (M.S.); florian.guisier@chu-rouen.fr (F.G.); luc.thiberville@univ-rouen.fr (L.T.); 3 Department of Cytology and Pathology, Rouen University Hospital, F-76000 Rouen, France; nicolas.piton@chu-rouen.fr

**Keywords:** bronchoscopy, peripheral pulmonary lesion, organizing pneumonia, radial endobronchial ultrasound

## Abstract

Background: The diagnosis of organizing pneumonia (OP) often requires histological confirmation. The aim of this retrospective study was to evaluate the diagnostic yield and complication rate of radial endobronchial ultrasound (r-EBUS) for OP. Methods: All patients who had r-EBUS as a first diagnostic procedure for a peripheral pulmonary lesion at Rouen University Hospital, France, between April 2008 and December 2020 were included. Cases without a final diagnosis of OP or follow-up were excluded. Patients, lesions, and r-EBUS characteristics were retrospectively analyzed. Results: 2735 r-EBUS procedures were performed, and 33 cases with final OP could be analyzed. Procedures were performed under local anesthesia in 28/33 cases (85%). Among the 33 final OP cases, 17 were considered cryptogenic, and 16 secondary. The lesions were patchy alveolar opacities in 23 cases (70%), masses or pulmonary nodules in 8 cases (24%), and diffuse infiltrative opacities in 2 cases (6%). A bronchus sign on CT scan was found in all cases. In 22 cases (67%), a histopathological diagnosis was obtained from the r-EBUS samples. In 4 cases (12%), histopathological diagnosis was made by surgery, and in 7 cases (21%) the diagnosis was made based on clinical, radiological, and evolution features. An ultrasound image was found in 100% (22/22) of cases in the r-EBUS positive (r-EBUS+) group vs. 60% (6/10) in the r-EBUS negative (r-EBUS-) group, respectively (*p* < 0.002). The diagnostic yield of r-EBUS for OP was 67% and increased to 79% (22/28) when an ultrasound image was obtained. The median time between CT scan and r-EBUS procedure was 14 days (3–94): 11.5 days in the r-EBUS+ group and 22 days in the r-EBUS- group (*p* < 0.0001). No severe complications were reported. Conclusion: r-EBUS, when performed shortly after a CT scan showing a bronchus sign, is an efficient and safe technique for OP diagnosis.

## 1. Introduction

Organizing pneumonia (OP) is a form of idiopathic diffuse interstitial lung disease (ILD). OP is characterized radiographically by variable features such as alveolar opacities (with a density varying from ground glass to consolidation), diffuse infiltrative opacities, and solitary mass (or nodule). The histopathological diagnosis is characterized by buds of granulation tissue within the distal air spaces (alveoli, alveolar ducts, and bronchioles), which are the hallmark of OP. OP may be associated with identifiable etiology including neoplasms, pulmonary infection, drug reaction, and connective tissue diseases, or may be considered cryptogenic, after exclusion of any other etiology [1]. Histologic confirmation is often required since the radiological abnormalities associated with OP are non-specific, with the main differential diagnosis being alveolar cell adenocarcinoma [1]. Furthermore, tissue samples may provide information on a possible cause of OP (especially infectious diseases and vasculitis). Surgical biopsy, which is the recommended procedure to obtain significant tissue sampling in ILDs, including OP, is associated with significant morbidity and mortality [2,3]. CT-guided lung biopsy is an acceptable alternative, showing an accuracy of up to 88% for OP diagnosis [4]. However, CT-guided biopsy is associated with a risk of pneumothorax ranging from 15 to 43%, and of pulmonary hemorrhage estimated between 1.0% and 27% [5].

Radial endobronchial ultrasound (r-EBUS) is a procedure using a 20 Mhz US probe that provides a high-resolution 360-degree view of the surrounding lung. Characteristic ultrasound features of normal and abnormal lung tissue help identify the lesion. After reaching the lesion, the probe is withdrawn while the guide sheath is left in place as an extended working channel. Biopsy forceps are then advanced through the guide sheath to the lesion to obtain tissue samples. In our center, r-EBUS is performed under topical anesthesia without sedation using a virtual bronchoscopy software to guide and shorten the length of the procedure [6], without fluoroscopy. Overall, for peripheral pulmonary lesions (PPL), the diagnostic yield of r-EBUS-guided transbronchial lung biopsy (TBLB) is higher (about 70%) than that of conventional TBLB without r-EBUS [6,7]. The reason behind the higher diagnostic yield of r-EBUS-guided TBLB appears to be the fact that it can accurately guide the probe toward the location of the pulmonary lesion. Moreover, fewer complications were observed with r-EBUS-guided TBLB than with unguided TBLB [8]. To date, only small case series have reported the use of r-EBUS for the diagnosis of OP in up to nine cases [9,10].

This paper describes results obtained in our department using r-EBUS and the virtual bronchoscopy procedure in OP. The aim of this study was to assess r-EBUS for the diagnosis of OP in a larger series of patients and to identify the factors affecting the diagnostic yield. 

## 2. Materials and Methods

### 2.1. Patients 

**Figure 1:**
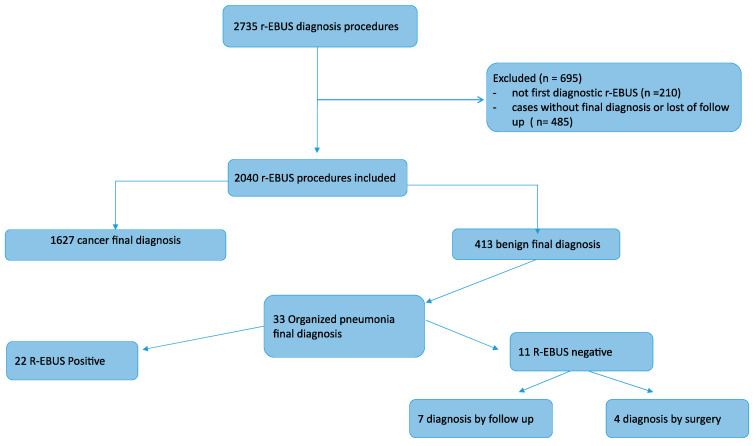
Flowchart.

We reviewed data from all patients who had r-EBUS-guided bronchoscopy for the diagnosis of a PPL, at Rouen University Hospital, France, between April 2008 and September 2020. From these, patients with a final diagnosis of OP were included in the analysis. (Figure 1) Diagnosis of OP was confirmed by histological sample when positive, or on the basis of clinical, radiological, and follow-up data when no histology was available. The patients were informed orally of the purpose of the endoscopic procedure, and the study protocol was approved by the Institutional Review Board for non-interventional research of Rouen University Hospital (protocol number E2021-20).

### 2.2. r-EBUS Procedure 

**Figure 2:**
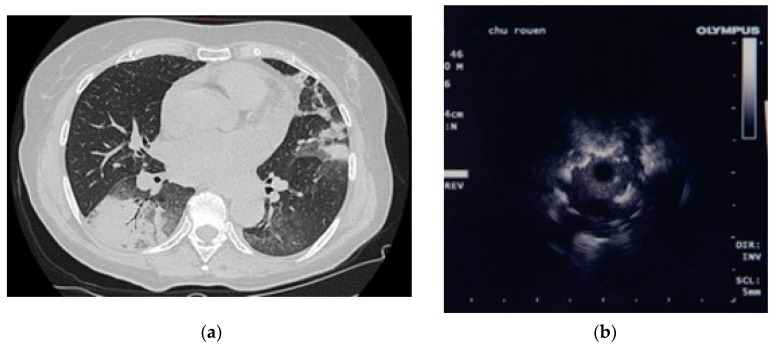
CT-scan image of organizing pneumonia with bilateral alveolar opacities (**a**). Ultrasonographic image of organizing pneumonia (**b**).

Before each r-EBUS procedure, the location of the lesion was analyzed using the virtual bronchoscopy software planner, in order to identify the optimal bronchial path to the lesion (Superdimension planner superDimension/Bronchus system, superDimension Ltd., Herzliya, Israel) from 2008 to 2011, and LungPoint®, Broncus Medical Inc., San Jose, CA, USA since 2011, from a millimeter (mm) slice chest CT (Figure 2a). A positive bronchus sign seen on a chest CT refers to the presence of a bronchus leading directly to a peripheral lung lesion.

A bronchoscopy was then carried out using local or general anesthesia with either the MP160F video bronchoscope (Olympus, Tokyo, Japan)or the BF-MP60F bronchofiberscope (Olympus, Tokyo, Japan) with a 4-mm outer diameter and a 2-mm working channel. The ultrasonographic (US) probe used was the 1.4 mm UM-S20-17S probe (Olympus, Tokyo, Japan), introduced into the dedicated 1.9-mm-diameter guide catheter (K201, Olympus, Tokyo, Japan). The procedure was performed without electromagnetic navigation or fluoroscopy. After having memorized the endobronchial route from the planning, the operator reached the most distal subsegmental bronchus. According to Izumo et al., the US image was a hypoechoic signal for patients with predominant alveolar/solid opacities and a mixed blizzard sign/hypoechoic signal for diffuse infiltrative opacities with GGos associated with solid components [11,12,13]. We were unable to find any differences between OP pattern images and other malignant images (Figure 2b).

**Figure 3:**
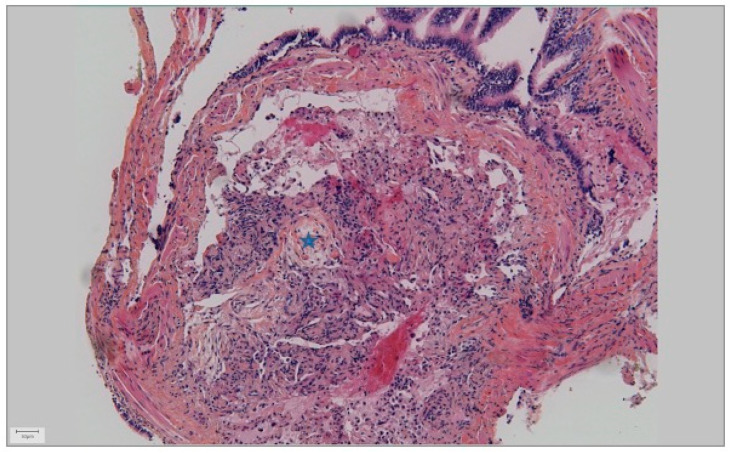
Pathology of organizing pneumonia (Star: buds of granulation tissue within the distal air spaces).

Once the US signal of the nodule was obtained, the probe was removed, and the guide sheath was left in place in the lesion. Sampling, including cytological brush and forceps biopsy (1.5 mm forceps from the dedicated kit K201, Olympus), was then performed. Bronchoalveolar lavage was also performed. Rapid On Site Examination (ROSE) was not used. Systematic chest radiographs were obtained after each procedure to ensure the absence of pneumothorax. Diagnosis was confirmed using the r-EBUS samples, when the histological hallmark of OP (buds of granulation tissue within the distal air spaces (alveoli, alveolar ducts, and bronchioles)) was found (Figure 3).

### 2.3. Statistics

Descriptive statistics were performed using Prism^®^ (GraphPad, San Diego, CA, USA). Comparisons were performed with a Fischer exact test or chi-squared test for qualitative parameters and a Student test for quantitative parameters. The tests were bilateral and the alpha risk was set to 5%. 

## 3. Results

### 3.1. Patients and Lesions 

Between April 2008 and December 2020, 2735 r-EBUS procedures were performed for PPL, 33 of which had a final diagnosis of OP. Among these 33 final OP diagnoses, 17 were considered cryptogenic, and 16 secondary (7 post infectious, 3 toxic due to immunotherapy, 3 due to malignant blood disease, 3 due to granulomatosis). All patients had clinical and radiological improvement: 20 (61%) with corticosteroids, 7 spontaneously, and 6 after surgical ablation.

On chest CT, the lesions appeared as patchy alveolar opacities in 23 cases (70%), masses or pulmonary nodules in 8 cases (24%), and diffuse infiltrative opacities in 2 cases (6%). They were bilateral in 24 cases (74%). A bronchus sign on CT scan was found in all cases. The median time between the CT scan and r-EBUS procedure was 14 days (3–94).

### 3.2. r-EBUS Procedures 

The r-EBUS procedure was performed under local anesthesia in 28/33 cases (85%). Bronchoalveolar lavage was performed in 18 cases (55%), showing an increased rate of lymphocytes in 14 cases (78%). Distal biopsies were performed through the guide sheath in 28/33 cases. A median number of four biopsies per patient was performed. No complications were reported during or after the procedure (pneumothorax or bleeding).

A US image was obtained using r-EBUS in 28 cases (85%). In 27 cases, the image was within the lesion and in one case adjacent to the lesion (Table 1). An example of an OP-related r-EBUS image is shown in Figure 2b.

The diagnosis of OP was obtained by r-EBUS biopsy sampling in 22/33 cases (67%), and in 22 of the 28 cases (79%) that were associated with an ultrasound image (*p* = 0.002). In four cases (12%), the histological diagnosis was obtained by surgery, and in seven cases (21%) the diagnosis was made by clinical, radiological, and follow-up data. 

The median time between the CT scan and r-EBUS procedure was 11.5 days in the r-EBUS+ group and 22 days in the r-EBUS- group (*p* = 0.0001).

## 4. Discussion

To our knowledge, the present work is the largest single center study that has evaluated the diagnostic yield, complications, and influencing factors of r-EBUS performance for organizing pneumonia.

In the present study, the diagnostic yield of r-EBUS for OP was 67%, and 79% when an r-EBUS signal was obtained during the procedure. No complications were observed during or after the procedure. 

A systematic review and meta-analysis found that the expected diagnostic yield for benign lesions using r-EBUS is around 60% (95% CI: 54.7–65.4%) [6]. A recent study by Kim et al. [8] compared the diagnostic yields of r-EBUS-guided TBLB to unguided TBLB in 127 patients with interstitial lung diseases. The diagnostic yield was 52.2% in the r-EBUS-guided group vs. 48% in the unguided TBLB group, with no statistical difference. 

r-EBUS has been previously described as a method for OP diagnosis in a very limited number of cases. In a study by Kim et al. [8], among 127 patients who underwent TBLB for ILDs, a diagnosis of OP was obtained in nine patients, seven of whom were diagnosed using r-EBUS. More recently, two small case series of six OP cases [9] and three OP cases [10] diagnosed using r-EBUS were reported in the literature.

On the other hand, series of OP explored by standard bronchoscopy with unguided TBLB [14,15] were reported by Dina et al. [14], who found that a histological diagnosis of OP on TBLB specimens was sufficient to confirm the clinical hypothesis in 7 out of 11 cases, and by Poletti [15] et al., who reported a (16/25) sensitivity of 64%. In these two studies, the authors did not provide information on complications. 

Overall, our study found a comparable diagnostic yield of r-EBUS for OP (67%, increasing to 79% when a US image was found) as reported using unguided TBLB [8,14,15], slightly superior to what is reported for other ILD. This could be explained by the fact that CT patchy alveolar opacities with a bronchus sign are more often present in OP compared to other forms of ILD [16]. In our study, a bronchus sign, a well-known factor associated with a high diagnostic yield of r-EBUS for PPL [6,17], was present in all cases. 

Recently, in order to increase the diagnostic yield for ILD, biopsy using a flexible cryoprobe was introduced in clinical practice. Metanalysis reported a 79% diagnostic yield of cryobiopsies. In a recent study, the authors reported a technique using r-EBUS for guided cryobiopsy without fluoroscopy [18], but with the same complication rate as the standard cryobiopsy technique. However, this study included only nine OP. Therefore, the superiority of cryobiopsy for the diagnosis of OP cannot be ascertained. 

In terms of complications, the rate of pneumothorax using r-EBUS-guided TBLB for the diagnosis of ILD [8] appeared lower compared to unguided TBLB. In addition to these complications, the most common method used to monitor the position of TBLB is fluoroscopy. 

In our study, a factor that may have influenced the diagnostic yield was the time delay between the CT scan and r-EBUS procedure. Indeed, we found that it was significantly shorter in the r-EBUS+ group than in the r-EBUS- group, which is possibly related to the high incidence of migratory alveolar opacities in OP [16,19]. These results suggest the importance of shortening the time between the CT scan and the sampling procedure, which may favor the use of a simple technique such as r-EBUS when performed under topical anesthesia without fluoroscopy. This is a retrospective and monocentric study. There is potentially a selection bias, as all OP were not referred for a bronchoscopy and, in our series, all of the patients had a bronchus sign.

As r-EBUS appears efficient, safe, and easy for the histological diagnosis of OP, this should be considered for every suspicion of OP, and moreover, for every consolidation pattern. The histological confirmation will help the physician to guide the steroid treatment, the therapeutic strategy in case of relapse, and search for an etiology. Future research should prospectively compare the non-invasive diagnostic techniques for OP, including r-EBUS, transbronchial biopsy (including transbronchial cryobiopsy), and trans-thoracic needle aspiration.

## 5. Conclusions

The results of our study have shown that r-EBUS, performed shortly after a CT scan showing the presence of a bronchus sign, is an efficient and safe technique for the diagnosis of organizing pneumonia, which compares favorably with standard TBLB.

## Figures and Tables

**Table 1:** r-EBUS procedures.

	r-EBUS + (22)	r-EBUS − (11)	*p*
CT scan patterns			
Alveolar opacities	16 (73%)	7 (64%)	0.7
Masse or pulmonary nodule	5 (23%)	3 (27%)	1
Diffuse infiltrative opacities	1 (4%)	1 (9%)	1
Bilateral/unilateral images	17 (77%)/5 (23%)	7 (64%)/4 (36%)	0.4
Median diameter (± IQR)			
Long axis (mm)	40 (9–80)	33 (14–79)	0.87
Short axis (mm)	20 (7–36)	20 (14–54)	1
Median (±IQR) nodule-to-pleura distance (mm)	0 (0–30)	(0–30)	
Bronchus sign	22 (100%)	11(100%)	1
Lesion location (%)			
Right upper lobe	2 (9%)	2 (18%)	1
Right middle lobe	1 (4%)	0 (0%)	
Right lower lobe	11 (50%)	4 (36%)	
Left upper lobe	3 (14%)	1 (10%)	
Left lower lobe	5 (23%)	4 (36%)	
US images	22 (100%)	6 (55%)	0.002
Invisible	0	5 (45%)	
Within the lesion	22	5	
Adjacent to the lesion	0	1	
Etiology of OP			0.6
Cryptogenic	12	5	
Secondary	10	6	
Median (±IQR) time between CT-scan and r-EBUS (Days)	11.5 days (1–17)	22 days (8–94)	0.0001

## Data Availability

The datasets used and/or analyzed in the current study are available from the corresponding author on reasonable request.
